# Supplementary material for: Isolation and Identification of an α-Galactosidase-Producing Lactosphaera pasteurii Strain and Its Enzymatic Expression Analysis
Source: Molecules. 2022 Sep 13;27(18):5942. doi: 10.3390/molecules27185942 (PMC9502112; doi:10.3390/molecules27185942)
Supplement: Supplementary file 1 [file molecules-27-05942-s001.zip › molecules-1909935-supplementary.pdf]

Figure S1. Sequences of *LpagaH* and its putative encoded amino acids

```
1     TAA TTC TTC TCT ATT GAG AGT GGA GAC AAC CCG TTC TGT CCC TAG   45
46    CGA TTG CGT TTC AAT ATA AGC ATG TGC AAA TAA ATT TAC GAG TAA   90
91    TTC ACC GAT GCT TTG GGC GTT TTT TCT GCT GAC AAC GGA TAA AGA   135
136   GTG ATA AAA TTC TTC GAG TGA TTT TCG TTC GCT TAT GGG TGC GTT   180
181   GTT TTT CGC AAC AAT ATC GCT GAT TAA TCC AAT ATC TCT TTG ATG   225
                                                               M   1
226   TCT AAA ACA GAG TTA GAA GGC ATT AAT GCC AAA CAA CAG ATG GTT   270
2      S   K   T   E   L   E   G   I   N   A   K   Q   Q   M   V   16
271   GAA CTG ATT AGA CAA AAC TTT AAT CAT CCG TCT ATC CTG TTC TGG   315
17     E   L   I   R   Q   N   F   N   H   P   S   I   L   F   W   31
316   GGA ATC CAG AAT GAA ATT CAA ATT AGT GGG GAA AGA CCC GAG CTA   360
32     G   I   Q   N   E   I   Q   I   S   G   E   R   P   E   L   46
361   AGA AAA CTC GTG AAT GAA TTA AAT GAG TTA ACG AAA AAG GAA GAC   405
47     R   K   L   V   N   E   L   N   E   L   T   K   K   E   D   61
406   CCT ACC CGA TTG ACT ACA ATG GCA AAC GTC ATG TTC GTT GAA GAT   450
62     P   T   R   L   T   T   M   A   N   V   M   F   V   E   D   76
451   GAA GAT GAC TAT AAT TAT GTG ACG GAT ACG ATT GGA TAC AAT AAA   495
77     E   D   D   Y   N   Y   V   T   D   T   I   G   Y   N   K   91
496   TAT TTT GGT TGG TAT AAT GGT GAA GCA GGG GAC TTT GCG GGC TGG   540
92     Y   F   G   W   Y   N   G   E   A   G   D   F   A   G   W   106
541   TTA GAT GGT TTC CAT AAG AAA AAT CCG ACT GTA AAA CTG GCG ATT   585
107    L   D   G   F   H   K   K   N   P   T   V   K   L   A   I   121
586   TCA GAA TAT GGT GCA GAA GGA ATT TTA CAA TAC CAT AGC AGT GAA   630
122    S   E   Y   G   A   E   G   I   L   Q   Y   H   S   S   E   136
631   CCA AAA ATA AAA GAC TAT TCT GAA GAA TAC CAT GCG CTT TAT CAT   675
137    P   K   I   K   D   Y   S   E   E   Y   H   A   L   Y   H   151
676   GAA ACC GTA TGG AAG ATT TTT GAA AAA CGT CCC TTC CTT TGG GCA   720
152    E   T   V   W   K   I   F   E   K   R   P   F   L   W   A   166
721   ACG TAT GCA TGG AAC ATG TTT GAC TTC GGT GCG AAT ATC AGA GAT   765
167    T   Y   A   W   N   M   F   D   F   G   A   N   I   R   D   181
766   GAG GGC GGT GTA CAG GGA AGA AAC AAC AAA GGG CTC ATC ACC TAT   810
```

Figure S2. Result of secondary structure prediction of α-galactosidase from *L. pasteurii* WHPC005

```
         10        20        30        40        50        60        70
          |         |         |         |         |         |         |
MSKTELEGINAKQQMVELIRQNFNHPSILFWGIQNEIQISGERPELRKLVNELNELTKKEDPTRLTTMAN
cccccccchhhhhhhhhhhhhcccccceeeecccccccccccccchhhhhhhhhhhhhccccccceeeee
VMFVEDEDDYNYVTDTIGYNKYFGWYNGEAGDFAGWLDGFHKKNPTVKLAISEYGAEGILQYHSSEPKIK
ccccccccccccccceeeeeeeecccccccccccchhhhhhhccccccceeeecccccccccccccccccc
DYSEEYHALYHETVWKIFEKRPFLWATYAWNMFDFGANIRDEGGVQGRNNKGLITYDRKIKKDAFYLYKA
ccchhhhhhhhhhhhhhhhcccccccccccchhccccccccccccccccccceeehhhhcchhhhhhccc
HWSDEKFVHITGKRFVDRTDDTIDIKVYSNCNEVNLSVNGGETTTLTSEDKIFVFENISLKEGINKVKVF
ccccccceeecccchhhhhcccccceeeecccccceeeeeccccccccccccceeeecccccccccceeeee
AKDGNTSLQDVAMFNKVDNPNESYFTPEEEGGFVANWFDMPELGNVEMEELVITDDVYSTRCTVGELFKN
ecccccccceeeeeeccccccccccccccccccceeecccccccccchhhhhheeeccccccceeehhhhhcc
EETKAIVTTYLGNVEEHPMFDMMQGFTIDAMSQIASDQFSEKMLYTLNKKLSQIKKSEQ
chhhhhhhhhhcccccccchhhhhhhhhhhhhhhhccchhhhhhhhhhhhhhhhhhccc

Sequence length :   409
```

Figure S3. Result of transmembrane region prediction of α-galactosidase from *L. pasteurii* WHPC005

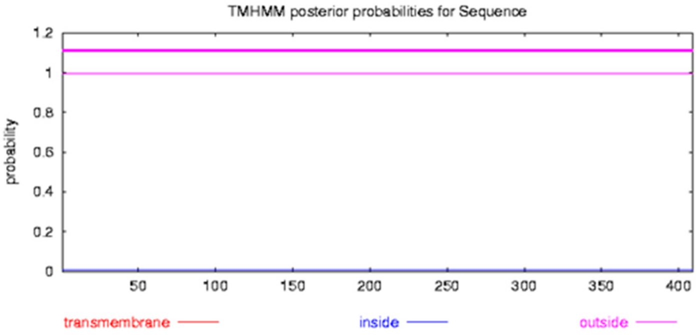

Table S1 Primers used in this study.

Primers	Sequences (5′→3′)	Usage
P1	AGAGTTTGATCCTG GCTCAG	To amplify the 16S rDNA for molecular identification
P2	ACGGTTACCTTGTTAC GACTT	
